# Digital Health Solutions for Weight Loss and Obesity: A Narrative Review

**DOI:** 10.3390/nu15081858

**Published:** 2023-04-12

**Authors:** Liam Irvin, Leigh A. Madden, Phil Marshall, Rebecca V. Vince

**Affiliations:** 1 School of Sport, Exercise and Rehabilitation Sciences, University of Hull, Hull HU6 7RX, UKphil.marshall@hull.ac.uk (P.M.); 2 Centre for Biomedicine, Hull York Medical School, University of Hull, Hull HU6 7RX, UK; l.a.madden@hull.ac.uk

**Keywords:** digital health, e-health, m-health, obesity, weight loss

## Abstract

Personal exercise programmes have long been used and prescribed for weight loss and the improvement of quality of life in obese patients. While individualised programmes are usually the preferred option, they can be more costly and challenging to deliver in person. A move to digital programmes with a wider reach has commenced, and demand has increased due to the SARS-CoV-2 pandemic. In this review, we evaluate the current status of digital exercise programme delivery and its evolution over the past decade, with a focus on personalisation. We used specific keywords to search for articles that met our predetermined inclusion and exclusion criteria in order to provide valuable evidence and insights for future research. We identified 55 studies in total in four key areas of focus, from the more recent development of apps and personal digital assistants to web-based programmes and text or phone call interventions. In summary, we observed that apps may be useful for a low-intensity approach and can improve adherence to programmes through self-monitoring, but they are not always developed in an evidence-based manner. Engagement and adherence are important determinants of weight loss and subsequent weight maintenance. Generally, professional support is required to achieve weight loss goals.

## 1. Introduction

It is widely recognised that overweight and obesity increase the risk of disease and mortality [1]. According to statistics from 2016, 39% of individuals aged 18 and above were overweight, and 13% were obese, resulting in more deaths than underweight individuals in most countries [2]. The most commonly used measure for weight by health organisations is the body mass index (BMI), which is a basic tool to classify groups of populations [3,4]. Statistics from Cancer Research UK reveal that, after smoking, overweight and obesity are the leading causes of preventable cancer in the UK [5].

The SARS-CoV-2 pandemic has had a significant effect on health and wellbeing, as evidenced by Zhu et al. [6]. The impact of mandates to remain at home alongside increased levels of stress and anxiety led to a significant increase in total food intake and a significant decrease in physical activity (PA), resulting in weight gain in many individuals. In addition, Okuyan and Begen [7] have pointed out that many businesses are in all probability looking to make work from home more routine, due to the economic consequences of SARS-CoV-2, the perceived convenience of less travel time, greater autonomy, and potentially reduced costs, for example, rental of large office spaces [8,9]. Consequently, there are more increased sedentary behaviours, including prolonged sitting, which evidence shows causes hypertension, musculoskeletal pain, and the potential for long-term health problems [10,11,12]. This increase in sedentary behaviour has led to reduced levels of PA [13], which in turn can contribute to the development of obesity. Regular or tailored exercise has been shown to have numerous health benefits that can help reverse the health problems associated with inactivity and obesity.

Digital technology has improved many aspects of modern-day life, from advancements in medical science to enhanced healthcare and outcomes in educational settings [14]. However, it is also associated with lessened attention, social isolation, and addiction, and has an impact on sleep [15].

In modern society, there is a requirement to use digital interventions to assist with health [16]. Digital health technologies enable remote monitoring of various health indicators, such as heart rate, diet, PA, and blood glucose levels in individuals with diabetes. They also improve diagnosis and treatment by providing a faster and more personalised service, information on symptoms, and advice [17], thereby reducing pressure on primary care services and physicians [18]. Furthermore, in a previous study [19], authors demonstrated a significant increase in energy expenditure per workday pre to post a digital prompt intervention to interrupt prolonged occupational sitting time and be able to utilise different technologies in a more workplace setting. Moreover, there is a desire from individuals to take advantage of these technologies if they are recognised as helpful and easy to use [20], and friends/family recommend them [21,22]. Therefore, these digital healthcare technologies are being increasingly used for weight loss in obese individuals, either in a generic manner or in a manner specifically tailored to the individual. The impact of weight loss is multifaceted, from a general health and wellbeing aspect to the economics of treatment when required.

This review aims to provide an up-to-date understanding of the digital health solutions for weight loss in overweight and obesity and to identify any potential gaps in this area.

## 2. Materials and Methods

### 2.1. Search Strategy

The database used for the literature search was Web of Science, using the following keywords: “digital health,” “digital intervention,” “weight loss,” “obes*,” “e-Health,” “m-Health,” “online weight management,” “online weight loss programme,” “web-based intervention,” “personal digital assistant.” Additional searches were conducted for specific platforms such as NHS, Slimming World, Cell Phone Intervention for You (CITY), MyFitnessPal (MFP), Weight Watchers (WW). The search was limited to articles published between 2011 and 2021 in the English language. We followed previously established frameworks for conducting a narrative literature review [23,24].

### 2.2. Study Selection

Inclusion criteria for the study were individuals aged 18 years or older with a BMI of 25 kg/m^2^ or higher, studies that used relevant keywords, mixed methods, process evaluation, randomised, experimental, controlled trial, and/or relevant methods/protocol with a control arm, and outcome measures related to weight loss or digital health. Exclusion criteria included study protocols alone, meta-analysis, systematic reviews, rapid reviews, and narrative reviews.

**Figure 1:**
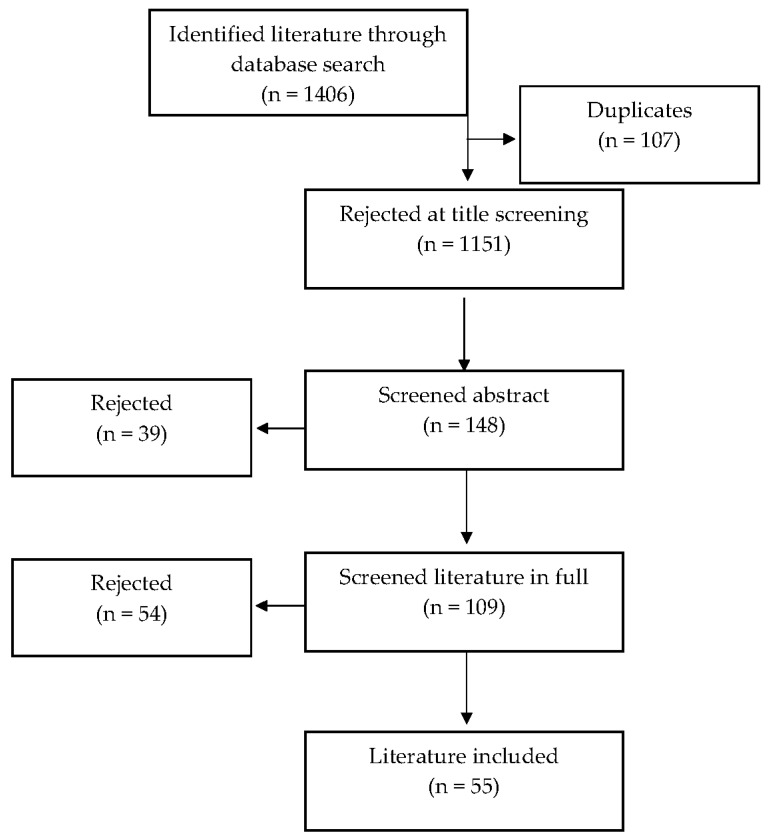
Flow diagram of identification, screening, and inclusion of studies.

The literature included was selected based on inclusion criteria, firstly by title, then abstract, and finally by full-text screening [25,26]. The initial search returned 1406 papers, with 107 duplicates and 1151 removed based on title (see Figure 1). From the 148 studies remaining, a further 39 were excluded after abstract screening due to irrelevancies. There were 109 studies remaining for full-text eligibility inspection; 54 were removed due to no control/no results. This left a total of 55 studies available for inclusion in the systematic review. The search strategy was formulated by the co-authors. Once the search was complete, the studies were listed in a spreadsheet, and the titles and abstracts were screened to identify potentially relevant studies. The full text was obtained for all studies that appeared to meet the inclusion criteria or where there was any uncertainty. Subsequently, two authors (L.I. and L.A.M.) examined each full-text manuscript to assess for eligibility, and neither was blinded to the journal titles or study authors, with any disagreement resolved through discussion or consultation with the third author (R.V.V.).

### 2.3. Data Analysis

As proposed by Fah and Aziz [27], articles were inserted into a tabular format to condense information and extract the most relevant information for review. The table included: study authors; participant information; intervention and control; and key outcomes. This step allowed for the differentiation of studies and their key themes.

## 3. Results

There were 55 studies out of 1406 results included in the systematic review (see Figure 1). From the available information, there were a total of 17,438 participants, aged 18–72 years, with a BMI of 25–50 kg/m^2^, and four studies had a higher percentage of male participants (57–100%).

### 3.1. Themes

Four key themes were derived from the literature, which included the use of an app, a personal digital assistant (PDA), web-based programmes, and text or phone call interventions.

#### 3.1.1. App

Table 1 shows the characteristics of those studies. In some cases, the authors were the designers and creators of the app (e.g., [6,28]), while others utilised readily available apps, such as MFP [29,30] and WW apps [31,32].

##### No Weight Loss Outcome Measure (Step-Count/Activity P/Week)

Several studies did not explore weight loss as an outcome measure; for instance, [46,56] used step count as their measure of PA. Mamede et al. [46] used a 10-week intervention consisting of different versions of an app in gamification, nudges, and the follow-up phase (four weeks). They reported participants increased steps/day by 634.0 ± 244.8 vs. control (*p* = 0.01) in phase one, but this did not continue in phase two (98.2 ± 325.5 steps/day, *p* = 0.76) or follow-up (53.49 ± 381.7 steps/day, *p* = 0.89). In Zhou et al. [56], the intervention (personalised steps/day based on past steps and goals) group decreased daily step count −390 ± 490 steps/day between run-in and 10 weeks vs. the control group (10,000 steps/day goal) decrease of −1350 ± 420 steps/day (*p* = 0.03); a between-group difference of 960 steps/day.

Another study examined the sitting time differences between weight-loss maintainers and weight-stable (obese) participants. Total weekly energy expenditure was significantly greater (*p* < 0.001) in the intervention group (1835 kcal/week) vs. control (785 kcal/week), and higher BMI was associated with increased sitting hours during weekdays and weekends (*p* < 0.001) [32].

Furthermore, Ainscough et al. [28] looked at dietary and PA interventions for overweight/obese pregnant women. The app group had healthy eating and exercise advice, whereas the control group received standard care (without advice). At 28 weeks, there was no significant mean difference in self-reported light PA (minutes/week) in the intervention vs. control (13.3 min/week; *p* = 0.111), but there was a significant difference in moderate activity in the intervention vs. control (26.5 min/week; *p* = 0.001) and an energy difference of −173.34 kcal in the intervention group vs. the control group (*p* < 0.001).

##### Non-Significant Weight Loss Outcomes

Allen et al. [33] investigated how the intensity of counselling may affect weight loss and change in PA; app only −1.8 ± 3.7 kg, IC −2.5 ± 4.1 kg, IC + app −5.4 ± 4.0 kg, less IC + app −3.3 ± 5.9 kg (*p* = 0.89); self-reported PA of moderate or greater intensity decreased in all groups bar app 0.19 ± 5.1 hrs/week; IC + app −2.0 ± 5.4 hrs/week; less IC + app −3.6 ± 5.5 hrs/week; IC −1.4 ± 7.1 hrs/week (*p* = 0.51). In a comparison of different self-monitoring and support groups vs. a no-monitoring control group, no significant differences in weight, body composition, or exercise were found between the groups [33].

Whitelock et al. [55] evidenced no significant findings between a food and drink attentive app vs. a dietary advice group. At eight weeks, weight loss for the app was 1.2 ± 3.1 kg vs. control of 1.1 ± 3.4 kg (*p* = 0.89), and body fat change at 8 weeks was −0.4 ± 1.8% vs. control of −0.5 ± 2.0% (*p* = 0.81). Brindal et al. [34] used an app with features including food intake recording, rewards, and prompts, but this was not a significant predictor of weight loss (6.67% in the supportive app group vs. 5.41% in the static app condition at 24 weeks (*p* = 0.36). Additionally, no difference for the interaction between study week and condition was found (*p* = 0.49). However, 58% of completers lost ≥5% of their body weight (between groups, *p* = 0.69), a clinically relevant amount of weight. Likewise, Duncan et al. [35] found no difference between a behaviour change app and an enhanced version and wait-list control in average body weight for the pooled intervention vs. control at 12 months (−0.00 kg). Moreover, daily energy intake was insignificantly lower in the pooled intervention than control at 12 months (−913.36 kJ), although this was significant at six months (difference −1037.03 kJ, *p* < 0.05). Hernández-Reyes et al. [38] found a non-significant difference in weight loss in a self-monitoring and push notifications app vs. no notification: −7.9 ± 3.9 kg vs. −7.1 ± 3.4 kg control (*p* = 0.39), but body fat loss was significantly increased in the intervention group (−12.9 ± 6.7%) vs. control (−7.0 ± 5.7%) (*p* < 0.001).

Forman et al. [31] reported that the main effect of treatment condition on percent weight loss was not significant (*p* = 0.70) between conditions WW and WW + OnTrack; however, diet type was an indicator of weight loss (*p* = 0.002). Gill et al. [37] compared the use of an app and allied coach for improving PA against a control (usual lifestyle), the between-group difference in weight was −0.46 kg (*p* = 0.63). However, the intervention increased step count (average 3,132 steps/day; *p* < 0.001) and decreased sitting time (−0.08 min/day; *p* = 0.03) between groups at 6 months. Hutchesson et al. [40] found differences for a targeted/tailored programme vs. wait-list in body fat in kg (−3.1 kg; *p* = 0.019), but not for body fat percentage (−2.0%; *p* = 0.093) or weight loss either measured or self-reported; although within-group weight, BMI, body fat (kg), and waist circumference were all significant in the intervention group. Additionally, a study tested an app in a gamification group given weekly targets, gamification plus physicians given data for feedback, or a basic 10,000 steps/day control. There were no significant differences in weight loss between interventions and control at any time point, but all groups lost significant weight at 12, 24, and 36 weeks [42].

Laing et al. [43] found MFP did not produce significant weight loss in overweight individuals, despite controls gaining weight at both time points. West et al. [54] investigated group-based weight loss via video and text conditions using MFP (−5.0 ± 6.0% vs. −3.0 ± 4.1%, respectively), and no significant self-reported levels of PA per week were observed between video (91.8 ± 107.4 min/week) vs. text (36.6 ± 35.7 min/week, *p* = 0.92). Monroe et al. [48] reported on participants’ self-monitoring using MFP and showed that weight loss did not significantly differ between groups (standard behavioural treatment, 5.30 ± 3.93% vs. enhanced (additional social support), 5.96 ± 5.19%; *p* = 0.63). There was no difference between groups for adherence to self-monitoring dietary intake (*p* = 0.37), or for the number of counselling sessions attended (*p* = 0.13).

##### Significant Weight Loss Outcomes

Hartman et al. [29] showed the impact of MFP and an accelerometer for individuals with elevated breast cancer risk, with significant reductions in weight −4.4 ± 4.3 kg vs. standard care 0.8 ± 3.8 kg (*p* = 0.004). Moderate-to-vigorous physical activity (MVPA) increased 15.01 ± 14.2 min/day in the intervention vs. 10.9 ± 10.1 min/day in usual care at 6 months, with the difference at 6 months being statistically significant (*p* = 0.02), although the difference between the changes in each group was not (*p* = 0.13). Johnson et al. [30] tested the impact of in-person (IP) vs. video conference (VC) health coaching and control. All groups used MFP and additional software, whereby weight loss was significantly greater (*p* < 0.05) for VC (8.23 ± 4.5 kg) vs. IP (3.2 ± 2.6 kg) and control (2.9 ± 3.9 kg). VC had 7054.6 ± 2068.7 steps/day at 12 weeks vs. control 5002.4 ± 2640.3 steps/day and IP 6236.2 ± 2393.4 steps/day (*p* < 0.05).

Fukuoka et al. [36] found a pedometer plus a self-monitoring/reminder app increased weight loss (−6.2 ± 5.9 kg) vs. a pedometer only (0.3 ± 3.0 kg; *p* < 0.001); and step count increased in the intervention to 2551 ± 4712 vs. the control’s −734 ± 3308 steps per day (*p* < 0.001). Moreover, Lugones-Sanchez et al. [45] compared a healthy diet and PA counselling against counselling plus app use. The intervention produced a greater loss of body weight −1.97 kg vs. control –1.13 kg (both significant to baseline *p* < 0.01, and between-groups, *p* < 0.01), with BMI between groups also significant (−0.54 kg/m^2^, *p* < 0.01). Muralidharan et al. [49] examined the impact of usual care and one nutritionist meeting versus an app and weekly calls. The reported change in weight in the intervention was −1.1 ± 3.0 kg (*p* < 0.01) vs. control 0.3 ± 2.9 kg (*p* < 0.05), with between-group differences observed for weight (*p* = 0.01), BMI (*p* = 0.002), and waist circumference (*p* = 0.01). In addition, Naimark et al. [50] found a weight change −1.44 ± 0.4 kg in the app group vs. −0.128 ± 0.36 kg in the control group (*p* = 0.03), alongside significant changes in BMI (−0.48 ± 0.13 kg/m^2^ vs. −0.03 ± 0.12 kg/m^2^ control; *p* = 0.03) and PA (63 ± 20.8 min/week vs. −30 ± 27.5 min/week control; *p* = 0.02). Mao et al. [47] utilised the Vida Health coaching programme and compared it to (pre-trial) historic weight data control. The matched-pair control group gained 1.81% total body weight (TBW) without Vida coaching and lost −2.47% with Vida, whilst the total intervention group lost 3.23% and 28.6% achieved a clinically significant weight loss of ≥5% TBW.

In [39] four groups were assigned either face-to-face, by app, in combination, or on the waitlist. Significantly more participants in all intervention groups had ≥5% weight loss vs. control, with more participants in the combination vs. app group losing ≥5% (19%; *p* = 0.06). Significantly reduced total energy intake was reported for all groups except the control. Another study [51] used three intervention groups and control, whereby the NHS weight loss plan and Slimming World did not significantly decrease weight loss compared to the control (−0.4 kg and −0.8 kg, respectively). However, Rosemary Online lost 1.5 kg more than the control (*p* = 0.001); 19% lost ≥5% in Rosemary Online, and <5 participants lost ≥10% weight in each group [51]. Lin et al. [44] found engagement measures were significant for weight loss at six months and personal coaching at 12 months, with no difference between groups for weight loss >5% at 24 months and large variability in weight loss in each arm. In [53], the app group (support, prompts) was not superior to control at any measurement point. Personal coaching (self-monitoring, group sessions) participants lost significantly more weight than controls at six months (−1.92 kg; *p* = 0.003), but not at 12 and 24 months. In the first six months, the app group self-weighed 4.0 ± 1.7 times/week vs. 2.2 ± 1.6 times/week in the personal coaching intervention.

Redman et al. [52] considered the impact of an app on reducing weight gain in pregnant women. Participation in a weight management programme either through an app or in-person (paper PA tracking) or usual care, whereby in-person had a mean gestational weight gain of 8.0 ± 1.3 kg vs. usual care 12.8 ± 1.5 kg (*p* = 0.04) and remote had a mean gestational weight gain of 10.0 ± 1.2 kg vs. usual care (*p* = 0.07). The proportion of women with excess gestational weight gain was significantly lower in person (56%; *p* = 0.03) and in remote groups (58%; *p* = 0.04) vs. usual care (84.6%).

#### 3.1.2. Personal Digital Assistant

Five studies used PDAs as their intervention; Table 2 shows the characteristics of those studies. These publications used the software Dietmate Pro, a dietary self-monitoring programme to track energy and fat intake, with the addition of CalcuFit [57], a PA self-monitoring programme. Each used two intervention groups: PDA or PDA + tailored feedback (TF) vs. a control group using self-monitoring via a paper record (PR). Three studies [58,59,60] were secondary analyses using data from Burke et al. [61]. 

##### Non-Significant Weight Loss Outcomes

Three studies found no between-group differences for weight loss, whereby [62] demonstrated the PR group had a percentage weight change of −5.94 ± 5.9% vs. PDA’s −6.71 ± 6.9% (*p* = 0.4), within-group *p* < 0.01; differences between groups were found in fruit and vegetable measures. Wang et al. [59] demonstrated percentage weight loss in the PR group of −5.19% vs. PDA −3.92% vs. PDA + FB −5.30% (*p* > 0.05); although self-monitoring was significant (PDA vs. PR; *p* = 0.014). Burke et al. [60] showed a significant within-group difference in weight over time in PDA + FB (average -2.32% weight loss; *p* = 0.02), but not for PR (−1.94%) or PDA (−1.38%), with non-significant between-group differences (*p* = 0.33).

##### Significant Weight Loss Outcomes

Although at 6 months, all treatment groups did have a significant weight loss (*p* < 0.01) with no significant differences among the groups (*p* = 0.12), Burke et al. [61] did find significant values for weight loss ≥5% in PDA + FB participants (63%) vs. PR 46% (*p* = 0.04) and PDA group 49% (*p* = 0.09). This is while Turk et al. [58] demonstrated that no daily feedback (PR and PDA) resulted in significantly less weight loss compared to feedback (PR + PDA −5.5 ± 6.2% vs. PDA + FB −7.3 ± 6.6%; *p* < 0.05).

#### 3.1.3. Web-Based

We identified 15 studies that applied web-based interventions, and these are summarised in Table 3.

##### No Weight Loss Outcomes Measure

Ballin et al. [66] investigated a web-based training programme (WE) compared to the same programme but in supervised groups at a clinic (SE), with the primary outcome being change in visceral adipose tissue (VAT) in a 50:50 male/female cohort. WE had no significant effect on VAT at 10 weeks compared to baseline 2025 ± 829 g (*p* = 0.5), and no between-group differences were observed for VAT (*p* = 0.11), although decreased fat mass was observed in WE (31,863 ± 5752 g (*p* = 0.034) and SE groups (32,353 ± 6004 g (*p* < 0.001) that did have between-group differences (*p* = 0.042).

##### Non-Significant Weight Loss Outcomes

In Rollo et al. [73], overweight/obese women with recent gestational diabetes enrolled in a web-based programme with either high personalisation, including video calls with a dietitian and exercise physiologist, or low personalisation with normal access to the programme, or wait-list control. At 6 months, the high-personalisation group lost 1.6 kg in comparison to the low-personalisation group, which lost 0.9 kg, and the wait-list control group, which gained 0.75 kg. No significant group-by-time effect was observed for weight when both treatment groups were compared against the control group (*p* = 0.137), no differences in MVPA were reported (*p* = 0.158); there was no significant reduction in diabetes risk at the group level. 

##### Significant Weight Loss Outcomes

A study compared four weight loss programmes, whereby significant weight loss was seen for all intervention groups in comparison to the control group: Curves Complete (CC) −4.32 kg, WW −4.31 kg, Jenny Craig −5.34 kg, and Nutrisystem −5.03 kg, with no difference between groups [65]. The control group gained 0.16 kg. Reductions in total energy intake were CC −413 kcal, WW −531 kcal, Jenny Craig −604 kcal, Nutrisystem −631 kcal, and control −103 kcal, with the largest reductions seen in Jenny Craig and Nutrisystem, both web-based interventions where meals were delivered [65]. Beleigoli et al. [67] investigated two interventions a web-based behaviour-change platform with tailored feedback vs. the platform plus additional coaching by a dietitian and a waiting list control with generic PA guidelines. Weight change at 24 weeks was higher using the platform plus additional coaching −1.57 kg vs. control, −0.66 kg (*p*< 0.001). Additionally, the platform only was 1.08 kg, whereby no difference was observed between intervention groups (*p* = 0.14). The intervention groups showed a higher proportion ≥5% weight loss, and longer usage of the platform was associated with clinically meaningful weight loss. Platform only and platform plus additional coaching both had improved dietary intake (increased consumption of fruits/vegetables and a reduction in ultraprocessed foods), but changes in PA did not differ between groups. Likewise, Collins et al. [68] found the intervention groups lost significant weight in a basic programme −2.1 ± 3.3 kg and the enhanced programme utilising personalised feedback and reminders lost −3.0 ± 4.1 kg vs. wait-list control weight gain of 0.4 ± 2.3 kg (*p* < 0.001). There were also significant between-group differences in the proportions of participants who lost 5 to <10% of their baseline weight (enhanced: 17%, basic: 18%, control: 3%; all *p* < 0.001). In Innes et al. [70], weight loss was significantly reduced post-intervention in all groups. The healthy weight loss programme achieved a weight loss of −5.17 ± 4.22 kg, compared to the free online NHS self-management weight loss tool of –4.19 ± 5.49 kg and gym only (no guidance) of –1.17 ± 3.00 kg; (*p* < 0.001). The two intervention programmes demonstrated greater reductions compared to gym only (*p* < 0.05). A separate paper found an adjusted difference of −3.16 kg (*p* < 0.0001) between WW and standard care control (−6.65 ± 0·43 kg vs. control −3.26 ± 0·3 kg, respectively) for those that completed the 12-month assessment, although at 12-months, 42% of participants had withdrawn from the trial [71]. This attrition differed significantly between countries (*p* < 0.0001), whereby the number of participants not completing in the UK (64%) was higher than in Australia (41%) and Germany (25%). Thomas et al. [75] investigated WW or WW plus additional behavioural training sessions. Both groups lost significant weight with no difference between groups at 3 months, whereas at 6 months weight loss in WW + ES was nearly twice as big as WW alone (4.7 ± 1.1 kg and 4.9 ± 1.3% vs. 2.6 ± 1.1 kg and 2.5 ± 1.3%, respectively, Ps < 0.047), highlighting the potential of behavioural change web-based VR technology as a tool to improve weight-loss outcomes to complement programmes, but requiring more investigation. 

Alencar et al. [63] identified a significant difference between video conference (VC; weekly specialist video) and the control group (baseline guidance) for body weight loss (−7.3 ± 5.2 kg vs. −1.2 ± 3.9 kg, respectively; *p* < 0.05); and for % body weight loss (−7.16 ± 4.4% vs. −1.5 ± 4.1, respectively; *p* < 0.05). Furthermore, 9/13 (69.2%) in the intervention group vs. 1/12 (8%) in the control group achieved a clinically significant weight loss of ≥5%. In addition, Alencar et al. [64] found weekly video calls (30 min) increased the healthy rate of weight loss significantly (−0.74 ± 1.8 kg) vs. the self-guided (baseline guidance) control group (0.18 ± 1.8 kg) (*p* < 0.05). Furthermore, it was reported in [76] that an accelerometer web-based intervention resulted in greater weight loss than a basic wait-list group. The intervention group lost −1.49 ± 0.26 kg in comparison to the control group (−0.82 ± 0.21 kg, *p* = 0.05), and BMI changes were reported as −0.50 ± 0.09 kg/m^2^ vs. control −0.29 ± 0.07 kg/m^2^ (*p* = 0.07). A subset analysis of “successful” intervention participants showed an 80% higher decrease in weight loss than the rest of the intervention group (*p* < 0.001) and that men were more successful at reaching the personalised targets than women. Additionally, Wijsman et al. [77] used the same intervention vs. wait-list, with findings showing significant weight loss between groups (intervention −1.49 ± 0.26 kg vs. control −0.82 ± 0.21 kg; *p* = 0.046) and waist circumference (−2.33 ± 0.36 cm vs. control −1.29 ± 0.34 cm; *p* = 0.036). The web-assisted PA intervention saw a significant increase in daily activity compared to the control alongside parameters of glucose metabolism and high retention rates, demonstrating the feasibility of use in an older population aged 60–70.

Conversely, weight loss was higher in the control group (Fitbit only) in [69] at six months −2.54 ± 4.00 kg (*p* = 0.002) vs. programme + Fitbit −1.71 ± 1.88 kg (*p* = 0.006) in breast cancer survivors. No significant difference was observed between groups (*p* = 0.461); three participants in each group achieved ≥5% weight loss. Similarly, Newlands et al. [72] explored a WW group, WW Plus (which included breast cancer-tailored support), and control, which received WW referrals at three months. Although significant weight loss was seen in both WW groups at 12 weeks (*p* < 0.001) that was not seen in the control group, despite the higher cost and time implications of running the extended programme the results showed no significance for 12-month weight change in WW Plus −1.22 kg (*p* = 0.436), but the WW and control groups did maintain at 12 months (−5.11 kg; *p* = 0.002 and −4.22 kg; *p* = 0.015, respectively). Thomas et al. [74] utilised WW online, or WWO, plus ActiveLink, using a PA tracking device and an informed control. All groups lost weight but no significant differences between groups in weight loss were observed at 12 months. At 12 months, more WW online participants (25.5%) lost ≥5% of their starting weight vs. controls (12.9%; *p* = 0.04), and neither group differed from WWO, plus ActiveLink (14.3%; *p* > 0.10) and more frequent engagement with the online platform was associated with superior weight loss.

#### 3.1.4. Tailored Text/Call

Six studies carried out tailored texts or calls as their measure, not including an app, as summarised in Table 4.

##### No Weight Loss Outcomes Measure

Wang et al.’s [82] study of 67 participants consisted of a group with a Fitbit One tracker + text message PA prompts vs. Fitbit One only in a short 6-week intervention. The SMS text-messaging effect lasted for a very short-term effect (1 week), whereby at baseline to week one, the intervention group increased by 1266 ± 491 steps vs. −48 ± 240 steps/day control (*p* = 0.01), MVPA 17.8 ± 8.5 min/week vs. 2.3 ± 4.1 min/week (*p* < 0.01) and 38.3 ± 15.9 min/week total PA vs. −6.7 ± 11.7 min/week total PA (*p* = 0.02) and afterward were insufficient in increasing PA. At the 6-week follow-up, there was a within-group significance (*p* = 0.04) in Fitbit only for MVPA of 4.3 ± 2.0 min/week and a between-group significance (*p* = 0.33).

##### Non-Significant Weight Loss Outcomes

Kim et al. [79] compared the effect of personalised text messaging to provide education and motivation, alongside additional offline education and counselling as a worksite weight management programme in comparison with standard care. They found no between-group differences at 6 months; the intervention lost −1.71 kg (*p* < 0.01) vs. control −1.56 kg (*p* < 0.001), no between-groups differences (*p* = 0.78) and no significant difference between groups for body fat percentage (*p* = 0.60) or PA min/wk (*p* = 0.14), although there was an overall positive perception of text messaging by participants. Further research found the impact of rewards via direct payment or lottery payment was no different from daily self-weighing and feedback alone (control) at 12 months: lottery vs. control −1.6 kg (*p* = 0.408); direct payment vs. control −0.5 kg (*p* = 0.813); lottery vs. direct −1.1 kg (*p* = 0.494), with the percentage of people who sustained weight loss in the lottery (66%), direct (62%), and control (59%) not significantly different (*p* > 0.1) [83]. As such, financial incentives provided no additional benefits for weight loss maintenance, but the group was likely highly motivated given their initial weight loss, coupled with additional systems attributable to success such as daily weighing, feedback, and ongoing participation in the WW programme and a trend was observed across arms that those who weighed themselves more frequently had greater weight loss (*p* < 0.001 at 6 and 12 months) [83].

##### Significant Weight Loss Outcomes

Fjeldsoe et al. [78] compared a tailored behaviour change text message intervention (‘Get Healthy, Stay Healthy’) to a standard control group. The intervention improvement in weight loss was significantly greater than controls (−1.35 kg; *p* = 0.003) and some forms of PA, but not in dietary behaviours. Another study utilised Facebook and text messaging to deliver either personalised (tailored) or generic (targeted) content vs. general healthy body weight content as a control in young adults with overweight/obesity [80]. Similar to other studies, engagement was an important determinant of weight loss, whereby a subset of highly engaged participants (completing >66% of the intervention) in the tailored group lost more weight compared to the control group at 6 months (−2.32 kg; *p* = 0.004) and 12 months (−2.28 kg; *p* = 0.04), although between-group differences disappeared by 8 months [80]. This was also shown by Steinberg et al. [81], who demonstrated that adherence was a key outcome whereby women who had ≥80% interactive voice response call completion rate had significantly greater weight loss of −1.97 ± 0.67 kg compared to those who achieved <80% (0.48 ± 0.69 kg; *p* = 0.01), and older, more educated participants were more likely to achieve a high interactive voice response call completion rate. Similar outcomes were also found for changes in BMI, and participants reported positive attitudes towards interactive voice response self-monitoring [81].

## 4. Discussion

The majority of studies used a weight loss of ≥5% of initial body weight as a measure of significant reduction. This value is generally accepted as a marker for health benefits in overweight/obese populations [84,85] and an indication of a strong intervention [86]. There may be an argument that there should be an aim for even greater losses, 5–10% within six months [87]. In contrast, Ryan and Yockey [88] state weight loss is individualised and <5 or >10% may be appropriate, depending on the physical state and health goals.

Data shows that step count can be used to test intervention effectiveness, in line with previous research that shows that step count is a viable and successful method of tracking and increasing PA in overweight populations [89,90]. Additionally, as the rise in wearable technology increases and with increasingly sophisticated technology (e.g., smartphones, smartwatches, smart rings), this offers more opportunities for people to access personal tracking capabilities for targeted physical activity goals. It’s suggested that pedometers lead to increases in PA and reduce risk in at-risk individuals without the need for additional counselling or incentives [90]. Further research (Mamede et al. [46]) has included gamification elements and social support features to increase step count in comparison to active control. They only found differences in the first five weeks of their study, which did not continue when physical nudges were used to promote the maintenance of behavioural changes achieved in the gamification phase.

Results indicate that tailored interventions can produce significant findings for weight loss and other health and PA-derived factors. Previous research found tailoring to be more effective in 66.6% (4/6) of studies in supporting weight loss than generic or wait-loss controls [91]. This is supported by [92], in which it was concluded that tailoring had a significant impact on digital interventions (*p* < 0.001) (see also [93]). The evidence, therefore, suggests there is a place within weight loss programmes for this alone or in addition to technology.

The present findings suggest that apps, in many cases, do not produce significant weight loss compared to controls. This resembles the evidence presented by Ghelani et al. [94], who concluded that apps may be useful as low-intensity approaches or alongside a weight management intervention to have the desired effect. In addition, the publications reviewed here have expressed that there needs to be an awareness that apps are not always developed on a solid evidence-base, and lack essential health aspects, so the addition of professional help may be required to attain weight loss goals [95,96,97]. Furthermore, a few studies tested the feasibility of an intervention plus an app/counselling. For example, Fukuoka et al. [36] found a pedometer plus the use of an app and in-person lifestyle intervention sessions resulted in greater weight loss than a pedometer alone in a diabetes prevention intervention in overweight adults. Equally, another study indicated that the addition of counselling via email increased PA compared to using a sole pedometer [98].

In this review, one of the most frequently used apps for remote recording of dietary intake and exercise for weight loss was MFP. It was shown to be advantageous in some cases, but as the lone intervention, no differences were found [43]. Some indicated MFP should be used more informatively while considering the security risks and safety measures of mobile health apps [99], while others have indicated the app provides precise values for elements of nutrition [100,101]. As the results of the current review suggest, it can be successfully utilised as a guide alongside additional software. For example, it has been used for dietary monitoring [30], whereas a Fitbit enabled accurate PA [29].

Five studies included the use of PDAs ([58,59,60,61,62]), whereby some weight loss was observed in interventions, but the majority of findings of the present study suggest PDAs are not considerably better than self-monitoring by PR, and additional feedback is not an improvement on PDA alone [102]. A review of technology in overweight and obese people found that the use of PDAs demonstrated an average weight loss of 2.0 kg, with PA monitors being the type of technology that achieved the greatest weight loss at 6.21 kg, followed by virtual reality (4.7 kg), websites (3.75 kg), smartphones (3.44 kg), and DVDs at the lowest (0.48 kg). This suggests that certain technologies can be effective to increase weight loss in patients with obesity while improving treatment adherence through self-monitoring [103]. Cavero-Redondo et al. [104] concluded, however, that smartphones were the most successful intervention when compared to web-based or PDAs for weight loss and adherence, and likewise, Khokhar et al. [105] found significant weight loss via mobile phone but little evidence for using PDAs vs. controls in promoting weight loss.

Three studies in the web-based theme found the programme alone produced increased weight loss over the programme plus an additional element compared to the control. Moreover, Nakata et al. [106] state the addition of a PA monitor resulted in no significant difference from self-help when looking at weight maintenance following weight loss in a web-based weight loss maintenance programme compared to self-help, although participants with greater increases in their step count lost more weight. A meta-analysis of eight studies found no significant difference between web-based and offline controls, although it reported significant heterogeneity between studies [107]. In contrast, a multitude of studies in the present review and Sorgente et al. [108] report that an enhanced intervention (i.e., one that is more interactive and tailored) contributes to greater weight loss. Furthermore, a review and meta-analysis of web-based versus offline interventions demonstrated the former resulted in greater short-term weight loss but not longer-term [109]. In contrast, Sorgente et al. [108] suggest uncertainty as to whether they are advantageous compared to comparable non-web-based programmes but they are better than a control/minimal intervention.

It was established that tailored text support resulted in greater differences in weight loss and PA outcomes. According to previous studies, long-term text-based interventions are an effective strategy for weight loss [110,111]. A review found significant weight loss/maintenance that could be a scalable intervention option, albeit with a limited understanding of long-term effects [112]. Meanwhile, Spark et al. [113] revealed positive long-term preservation on weight and PA levels using text messages after weight loss intervention. In addition, an evaluation stated that participants find texts and call support a useful intervention, maintaining motivation and encouragement during interventions as a simple and convenient mode of communication [114]. Whilst we present a comprehensive review of the current state of the literature, a limitation here is the exclusive use of studies presented in the English language. There may be pertinent studies published in other languages. Furthermore, there is heterogeneity between studies with a large diversity in population characteristics in terms of age, gender, specific conditions, various platforms/modes of delivery that have considered many different components, from the most basic to the most in depth. These then require varying levels of commitment from participants, alongside differences in programme duration, how much human interaction/cost is required, and also various durations in follow-up time for assessment of weight maintenance, so that it is then difficult to directly compare programme efficacy and success therein. However, given the numerous barriers to exercise and physical activity in obese and clinical populations, digital platforms offer some way into a solution to tackle this given they have the potential to provide convenient and equitable access with longer-term tracking capability. However, this also needs to consider the personal preferences of the individual in terms of how they want to engage with technology and the level of human interaction and support required, whether social elements are required, and behavioural change techniques to prevent weight regain, as it is clear that engagement and adherence are important determinants of weight loss. What is also not considered is whether people paying for access to digital platforms subsequently affect user adherence, engagement, and success, either positively or negatively, and the longer-term use of digital health technology needs to be assessed. The increase in digital platforms also needs to be considered more from the privacy and security of consumers’ personal and health information [99]. Nevertheless, the possibility of positively using digital health solutions for obesity and weight loss is clear overall, and future developments should also look to tackle the management of specific conditions alongside weight loss and allow for a more individualised prescription of exercise.

## 5. Conclusions

There are many uses for digital interventions in addition to weight loss and maintenance. This narrative review was intended to examine the expanding digital health solutions to weight loss in overweight and obesity and has introduced contemporary evidence of the developments in ‘digital health’ and what to expect moving forward, for instance, enhanced apps that cater to individualised needs, alongside strategies to promote adherence and motivation to accompany an increase in the utilisation of these tools. The use of technology at present appears to work well, but individuals also require specialist support to achieve their weight goals and then subsequent weight maintenance.

## Figures and Tables

**Table 1:** Study characteristics for the use of an app.

Year/ Study	Participants	Digital Health Solution	Outcomes
2020 [28]	*n* = 565 Intervention aged 32.64 ± 4.60 years; BMI 29.44 ± 3.60 kg/m^2^ Control aged 32.22 ± 4.23 years; BMI 29.07 ± 3.28 kg/m^2^ 100% female	Intervention—smartphone app, nutrition, healthy eating and exercise advice, fortnightly emails Control—usual care, not including dietary advice	No significant difference in light activity intervention vs. control (*p* = 0.111) Moderate activity intervention vs. control (*p* = 0.001)
2013 [33]	*n* = 68 aged 44.9 ± 11.1; BMI 34.3 ± 3.9 kg/m^2^ 78% female	App—Lose it! Intensive counselling (IC; Month 1 counselling weekly, Months 2–6 biweekly) Intensive counselling + app (IC + app; Month 1 counselling weekly, Months 2–6 biweekly) Less intensive counselling + app (less IC + app; Month 1 counselling twice; then monthly counselling for months 2–6) Control—App (one counselling session)	No significant difference between groups for any outcome—change in body weight *p* = 0.89; BMI *p* = 0.79; PA *p* = 0.51; dietary intake (kcal/day) *p* = 0.66
2018 [34]	*n* = 146 aged 48.11 ± 11.75 years; BMI ≥25 kg/m^2^ 71% female	Intervention—Weight Management Programme (WMP; an app with a meal replacement programme; prompts; face-to-face support) Control—control/static app with no recording tools or any tasks; programme information, including recipes	Percent weight change from baseline at 24 weeks – Intervention 6.67% vs. control 5.41%; no difference in weight by different app condition (*p* = 0.36), or interaction between week and app condition (*p* = 0.49)
2020 [35]	*n* = 116 aged 44.5 ± 10.5 years; BMI 31.7 ± 3.9 kg/m^2^ 74% female	App—Balanced Traditional—access to app, PA, diet tracking, and feedback Enhanced—access to the app, PA, diet, sleep tracking, and feedback Control—waiting list	Insignificant weight change – Pooled intervention 6 months between-group difference −0.92 kg; 12 months −0.00 kg Intervention group significantly increased resistance training reporting to guidelines (*p* = 0.041) and reduced energy intake −1037.03 kJ/d vs. control at 6 months, but not at 12 months
2019 [31]	*n* = 181 aged 46.29 ± 13.58 years; BMI 34.32 ± 5.65kg/m^2^ 85% female	Intervention—WW + OnTrack; algorithm predicts lapses, prompts to update the state of 17 potential lapse triggers Control—WW daily dietary “Smartpoints” goal to achieve negative energy balance and weight loss, midway from Beyond the Scale (BTS) to a more flexible programme (Freestyle)	Effect of treatment condition on percent weight loss not significant (0.15; *p* = 0.70) Among BTS participants, weight losses were greater for WW + OnTrack (mean = 4.7%, SE = 0.55) vs. WW (mean = 2.6%, SE = 0.80). This pattern was reversed among Freestyle participants—WW + OnTrack (mean = 2.9%, SE = 0.38) vs. WW (mean = 4.5, SE = 0.52)
2015 [36]	*n* = 61 aged 55.2 ± 9.0; BMI 33.5 ± 6.0 kg/m^2^ 77% female	Intervention—mobile phone-based diabetes prevention programme (mDPP) + pedometer (self-monitoring of weight, activity, and caloric intake with daily reminders) + six in-person sessions Control—Pedometer only (no specific step goals were provided)	Intervention −6.2 ± 5.9 kg weight loss between baseline and 5-month follow-up vs. control gain 0.3 ± 2.7 kg (*p* < 0.001) Intervention increased daily step count by a mean of 2551 ± 4712 steps/d vs. control −734 ± 3308 steps/d (*p* < 0.001)(38%), between baseline and 5-month follow-up Intervention significant increase in reported PA (*p* = 0.03); no effect on self-reported total calorie/fat intake; significantly greater reductions in saturated fat intake than controls (*p* = 0.007) and sugar-sweetened beverage consumption (*p* = 0.02)
2019 [37]	*n* = 118 Intervention—aged 56.8 ± 12.3 years; BMI 32.0 ± 9.3 kg/m^2^ Control—aged 58.6 ± 14.7 years; BMI 30.9 ± 7.3 kg/m^2^ 79% female	Intervention—Three phases: the active phase (months 0–6)—meeting with a coach every other month with access to a suite of e-Health technology supports. Followed by a minimally supported phase 1 (months 6–12)—access to eHealth tools and resources, but no coaching. Followed by minimally supported phase 2 (months 12–18)—access to the HealtheSteps app and website) Control waiting list, provided publicly available resources related to healthy lifestyles	Between group differences at 6 months Intervention—step count increased by 3132/day (*p* < 0.001) more than the control group (N.b. intervention group increased by 1646/day and control group decreased by 1485 steps/day). Sitting time decreased (mean = −0.08 min/day (*p* = 0.03)). No differences in weight or waist circumference were observed between groups at 6 months Outcomes were maintained at minimally supported phase one; phase two retained improvements in sedentary time and healthful eating.
2016 [29]	*n* = 54 with increased breast cancer risk aged 59.5 ± 5.6 years; BMI 31.9 ± 3.5 kg/m^2^ 100% female	Intervention—MFP + Fitbit (12 × 30 min standardised coaching phone calls with trained counsellors) Control—usual care (US dietary guidelines) and two 15 min goal-setting calls, months 2 and 5)	Intervention participants lost significantly more weight (4.4 kg vs. 0.8 kg, *p* = 0.004) and a greater percentage of their starting weight (5.3% vs. 1.0%, *p* = 0.005) than usual care participants Moderate-to-vigorous PA increased in the intervention group by 15.01 min/day (SD = 14.2) vs. 10.9 min/day (SD = 10.1) in the usual care group. The difference at 6 months was significant (*p* = 0.02), but the difference between the changes in each group was not (*p* = 0.13)
2020 [38]	*n* = 60 aged 41.5 ± 11.3 years; BMI 31.8 ± 5.3 kg/m^2^ 100% female	App—Nutrición Sur Intervention—received Push notifications—exclusive access to specific functionalities of the app and push notifications Control—no access to functionalities related to the self-monitoring of weight at home, gamification, and prescription of PA Both groups followed the same diet. The women in the intervention and control groups were randomly assigned to programmes of PA of different intensities—light, moderate, and intense	Receiving notifications during the intervention increased body fat loss (mean = −12.9 ± 6.7% intervention vs. −7.0 ± 5.7% control (*p* < 0.001)) and helped to maintain muscle mass −0.8 ± 4.5% in the intervention vs. −3.2 ± 2.8% in the control (*p* = 0.018) Between groups, there was an insignificant difference in weight loss (−7.9 ± 3.9 kg in the intervention group vs. −7.1 ± 3.4 kg in the control group (*p* > 0.05))
2018 [39]	*n* = 81 aged 18–65 years; BMI > 29 kg/m^2^ 69% female	Conventional—face-to-face weight loss programme Mobile weight loss app (b-SLIM app)—digital advice for dietary pattern, PA, behavioural, monitoring, information, and support elements Partial conventional or partial mobile weight loss programme (Combi group)—initial dietitian advice/PA coach (same information as the conventional treatment group) and follow-up with a PA coach + use of the mobile weight loss app Control—waiting list for full programme	Significantly more participants in all three intervention groups lost at least 5% or more of their weight at baseline compared to the control group. More participants in the Combi group lost 5% or more compared with the app group (19%, *p* = 0.06). There is no significant difference between the Combi group and the conventional group All intervention groups had significantly higher decreases in cardiometabolic risk factors compared with the control group (all *p* < 0.05), but no significant differences were found between groups Significantly reduced total energy intake in the conventional group, app group (*p* = 0.001), combi group (*p* < 0.001), but not in the control group (*p* = 0.22)
2018 [40]	*n* = 57 aged 27.1 ± 4.7 years; BMI 29.4 ± 2.5 kg/m^2^ 100% female	Intervention—Be Positive Be Healthe (BPBH) e-Health technologies only, comprising five delivery modes (website, app, email, text messages, and social media) Control—waiting list for full programme	No significant differences were found in weight loss Significant mean differences for the intervention group for body fat −3.10 ± −5.69 kg (*p* = 0.019); intake of vegetables (% energy/d) 4.71 ± 2.20 (*p* < 0.001); intakes of alcohol (g) −0.69 (*p* = 0.037); and energy-dense, nutrient-poor foods (% energy/day) −9.23 (*p* = 0.018)
2019 [30]	*n* = 30 In-person (IP) aged 42.2 ± 10.2 years; BMI 35.3 ± 5.2 kg/m^2^; Videoconferencing (VC) aged 43.0 ± 10.7 years; BMI 38.6 ± 9.8 kg/m^2^; Control aged 44.5 ± 12.1 years; BMI 34.5 ± 5.3 kg/m^2^ No gender information	Apps—Withings HealthMate, Healow, and MFP IP and VC—individualised health coaching by a multidisciplinary team (registered dietitian, exercise physiologist, and medical doctor) Control—no coaching or feedback	Weight loss was significantly greater for VC (8.23 ± 4.5 kg) than IP (3.2 ± 2.6 kg) and control (2.9 ± 3.9 kg) (*p* < 0.05); there was a significant difference in steps at 12 weeks in the VC group vs. IP and control (*p* < 0.05) There were no differences in BMI between groups or between intervention groups and control
2017 [41]	*n* = 250 aged >18 years; BMI ≥ 27 kg/m^2^ 62% female	5 groups: (1) Daily weighing—self-weighing at the same time every day, monthly personalised feedback and encouragement (2) MFP group—track dietary intake every day for the first month using the app or website; one week every month during months 2–12 (3) Brief support—10–15 min monthly individual meetings, weighing and discussing ongoing successes and challenges (4) Hunger training—test blood glucose for the first two weeks before eating and eat or retest depending on if their blood glucose was less than or equal to their individualised cutoff, and complete the hunger booklet for one week every month after month one (5) Control—not provided with any monitoring strategies All participants (including those in the control group) could choose one of three possible dietary plans (Mediterranean diet, Paleo diet and Intermittent fasting) and one of two exercise programmes they wished to follow	At 12 months, there were no significant differences in weight, body composition, blood markers, exercise, or eating behaviour between the four monitoring groups and the control group (*p* ≥ 0.053)
2018 [42]	*n* = 196 aged 41.4 years; BMI 36.3 kg/m^2^ 86% female	Study participants formed teams of two with a family member or friend App—Withings HealthMate Gamification—weekly weight targets over 24 weeks, which continued through 36 weeks with an updated weight target; Gamification + primary care physician (PCP) data sharing—weekly weight targets over 24 weeks, which continued through 36 weeks with an updated weight target and weight and step data shared regularly with each participant’s PCP regularly for 36 weeks Control—app (goal of 10,000 steps/day)	Significant mean weight loss from baseline to 24 weeks in the control arm (−3.9 lbs), gamification (−6.6 lbs), and gamification + PCP (−4.8 lbs) (all *p* < 0.001); At 36 weeks, weight loss from baseline remained significant in the control group (−3.5 lbs, *p* = 0.01), gamification group (−6.3 lbs, *p* < 0.001), gamification + PCP group (−5.2 lbs, *p* < 0.01). In the main adjusted model, there were no significant differences between groups. There were no significant differences in the interventions vs. control in step count (gamification *p* = 0.24 and gamification + PCP *p* = 0.91) at 24 weeks or 36 weeks
2014 [43]	*n* = 212 primary care aged 43.3 ± 14.3 years; BMI 33.4 ± 7.09 kg/m^2^ 73% female	Intervention—usual primary care + MFP with instructional video Control—usual primary care, were told to choose any activities they’d like to lose weight and were blinded to the name of the app	Intervention group lost −0.06 lbs at 3 months vs. the control group, who gained 0.54 lbs, with no significant difference between groups (*p* = 0.53) At 6 months, the intervention lost −0.07 lbs vs. the control group, who gained 0.60 lbs (*p* = 0.63) There was no significant difference between groups in weight change −0.67 lb (*p* = 0.63)
2018 [44]	*n* = 365 aged 29.3 ± 4.2 years; BMI 35.3 ± 7.9 kg/m^2^ 70% female	Intervention—CITY app, with 24 components, including weight tracking and prompts by the app in predetermined frequency and forms Personal coaching—6 group weekly, two-hour group sessions conducted by an experienced coach with registered dietitian training, followed by 21 monthly phone coaching calls. Personal coaching participants were encouraged to use the CITY app to track weight, diet, and PA for monthly discussions Control—handouts on healthy eating and exercise for weight management	Engagement in the CITY intervention was associated with weight loss during the first 6 months, although engagement substantially dropped early on for most intervention components Engagement correlated to weight loss for both interventions at 6 months. This continued in the personal coaching arm for 12 months, but not in the app group at 12 months Weight loss >5% 24 months, no difference between groups
2020 [45]	*n* = 440 Intervention—aged 47.4 ± 10 years and BMI 32.8 ± 3.3 kg/m^2^; Control—aged 48.8 ± 9.2 years and BMI 32.9 ± 3.4 kg/m^2^ 70% female	App—EVIDENT 3 Intervention—app and smart band, a healthy diet and PA counselling Control—healthy diet and PA counselling	Intervention group achieved greater weight loss −0.84 kg more than the control at 3 months (*p* < 0.01). A significant between-group difference was noted only in BMI after the intervention (−0.54 kg/m^2^ more in the intervention than in the control (*p* < 0.01))
2021 [46]	*n* = 234 Intervention aged 47.5 ± 9.6 and BMI 26.9 ± 5.05 kg/m^2^; Control aged 45.9 ± 10.2 years and BMI 25.6 ± 4.5kg/m^2^ 62% female	App—MoveMore Intervention—10 week multicomponent intervention on PA and sedentary behaviour of office workers. Five week gamification phase with social support features and a five week physical nudges phase gamified digital app Control—basic version of the app (self-monitoring, goal setting)	Gamification stage intervention increased steps/day vs. control (*p* = 0.01). These improvements were not sustained during the physical nudges phase (*p* = 0.76) or follow-up (*p* = 0.88)
2017 [47]	*n* = 1012 aged 44.6 ± 11.3 years; BMI 33.5 ± 0.21 kg/m^2^ 67% female	Intervention—Vida Health app (4 months of intensive health coaching via live video, phone, and text messages) Control group—historic weight data prior to starting programme	Intervention group: −3.23% total body weight (TBW) at 4 months In the matched-pair control group, participants gained 1.81% TBW at 4 months without Vida vs. −2.47% TBW after 4 months with Vida coaching Intervention participants (28.6%) achieved a clinically significant weight loss ≥ 5% TBW, with an average 9.46% weight loss in this cohort
2019 [48]	*n* = 33 aged 44.67 ± 8.96 years; BMI 36.22 ± 7.53 kg/m^2^ 100% female	Intervention—ENHANCED (programme + two additional digital scales and Fitbit Zip to share with up to two adults in their existing social network) Control—standard treatment (technology-supported behavioural weight-loss treatment, Fitbit Zip)	Average weight losses from baseline to 16 weeks did not significantly differ between groups (*p* = 0.63) No significant difference in control vs. intervention for the mean number of days of self-monitoring of dietary intake during treatment or follow-up, or the number of counselling sessions attended over the intervention between groups
2021 [49]	*n* = 561 Intervention aged 37.8 ± 9.2 and BMI 29.4 ± 3.8 kg/m^2^; Control 37.8 ± 9.6 years and BMI 29.3 ± 4.2 kg/m^2^ 43% female	Intervention—mobile health and diabetes (mDiab) programme consisting of video lessons, SMS, infographics, and weekly health coach calls Control—usual care, consultation with nutritionist, diabetes prevention handouts for increased PA and weight loss	mDiab group had a small reduction in waist circumference compared to the control group (*p* < 0.01) There were significant between group differences in weight loss (*p* = 0.01) and BMI (*p* = 0.002). There was no significant difference in % body fat between groups (*p* = 0.48)
2015 [50]	*n* = 85 aged 47.9 ± 12.3 years; BMI 26.2 ± 3.9 kg/m^2^ 64% female	Intervention—eBalance Web-based app to monitor dietary intake and PA by receiving real-time feedback and healthy lifestyle presentations and nutrition/PA recommendations Control—healthy lifestyle presentation and nutrition/PA recommendations, and then instruction to continue with lifestyle	Significant differences in app group vs. control were found for weight (*p* = 0.03), BMI (*p* = 0.03), knowledge score (*p* = 0.04), and PA (*p* = 0.02). There was no significance in waist circumference (*p* = 0.09) App frequency of use was significantly related to a higher success score (*p* < 0.001)
2021 [51]	*n* = 528 aged 51.0 ± 15.0 years; BMI 35.8 ± 5.1 kg/m^2^ 63% female	NHS Weight Loss Plan—freely available NHS website (no time limit) Rosemary Online—access to an online coach via chat function (8 weeks) Slimming World Online—access to an online support team via the chat function on their website (3 calendar months) Control—no contact until final weight measurement	On average, all groups lost weight over the course of the study Only the Rosemary Online group showed a significantly greater weight loss compared with the control group (*p* < 0.001), losing 1.5 kg more than the control group and being more than three times more likely to have lost ≥5% of their body weight during the initial 8 weeks vs. the control group NHS and Slimming World weight loss is not significantly different from the control In each group, ≤5 participants lost ≥10% body weight
2017 [52]	*n* = 54 pregnant women aged 18–40; BMI 25–40 kg/m^2^	Remote—SmartMoms intensive intervention delivered either through (1) mobile phone (remote group) or (2) traditional in-person, clinic-based setting (in-person). Intervention included self-monitoring weight, activity tracking, personalised dietary intake, receipt of health information and feedback from counsellors Control—usual care of their obstetrician, no weight management services	Intervention was effective at reducing gestational weight gain (usual care 12.8 ± 1.5 kg, combined intervention 9.2 ± 0.9 kg (*p* = 0.04)) In-person group gained significantly less total weight during pregnancy than the usual care group (*p* = 0.04) Adherence was greater in the remote vs. in-person group (76.5% vs. 60.8%, *p* = 0.049)
2021 [32]	*n* = 5603 Intervention aged 53.9 ± 12.7 years and BMI 27.7 ± 5.4 kg/m^2^; Control aged 47.1 ± 13.0 years and BMI 39.2 ± 7.6 kg/m^2^ 90% female	Intervention—Weight-loss maintainers (WLM) with self-reported WW modalities (group meetings, website, mobile phone) used to lose weight and maintain weight loss Control—Weight-stable individuals with obesity (past or current WW membership with a BMI ≥ 30 kg/m^2^)	Weekly energy expenditure was higher in the intervention (*p* = 0.0001) Weight-loss maintainers expended three times more calories in moderate-to-vigorous PA (678 kcal/week vs. 182 kcal/week, respectively (*p* = 0.0001)) Greater correlations exist for weight maintainers between BMI and sitting hours during weekdays and weekends than for controls with weight-loss maintainers sitting less
2015 [53]	*n* = 365 aged 29.4 ± 4.3 years; BMI 35.2 ± 7.8 kg/m^2^ 70% female	App—Cell Phone Intervention for You (CITY) Cell phone intervention—smartphone used for both intervention delivery and self-monitoring, goal setting, and support Personal coaching intervention—delivered primarily by an interventionist and delivered during six weekly group sessions, followed by monthly phone contacts and a smartphone used exclusively for self-monitoring and shared with the interventionist for coaching sessions Control—three handouts on healthy eating and PA	Cell phone intervention was not superior to control at any measurement point Personal coaching intervention had the greatest mean weight loss and significantly more weight loss vs. control at 6 months (*p* = 0.003), but not at 12 and 24 months Personal coaching had greater weight loss than a cell phone at 6 months (−2.19 kg, *p* < 0.001) and 12 months (−2.10 kg, *p* = 0.025) There were no significant differences in weight loss at 24 months among the treatment groups
2019 [54]	*n* = 32 aged 47.2 ± 12.4 years; BMI 34.1 ± 5.5 kg/m^2^ 100% female	Video—weekly group chat sessions and cellular-enabled scale (video) Text-based—weekly chat sessions and digital scale (text)	There were no significant differences in weight loss and self-reported PA between conditions Video group had higher levels of engagement in chat sessions (62%) vs. text-based (50%), with no significant difference between groups
2019 [55]	*n* = 107 Intervention aged 42.8 ± 10.5 years and BMI 35.9 ± 6.8 kg/m^2^; Control aged 44.5 ± 10.7 years and BMI 35.2 ± 6.2 kg/m^2^ 74% female	App—Attentive Eating Intervention—App + standard dietary advice booklet and weekly advice text message Control—standard dietary advice booklet and weekly advice text message	There was no significant difference in weight loss between groups, weight change at 8 weeks (*p* = 0.89), or self-reported energy intake at 8 weeks (*p* = 0.67) Adherence to the intervention did not predict weight change (*p* = 0.15)
2018 [56]	*n* = 64 aged 41.1 ± 11.3 years; BMI 27.3 ± 6.1 kg/m^2^ 83% female	App—CalFit iOS app Intervention—app (automated personalised daily step goals) Control—app (fixed daily step goals 10,000 steps/day)	Intervention had a lesser decrease in steps/day between the run-in period and 10 weeks vs. control (*p* = 0.03)

**Table 2:** Study characteristics for the use of a PDA.

Year/ Study	Participants	Digital Health Solution	Outcomes
2011 [62]	*n* = 192 aged 49 years; BMI 34.0 ± 4.5 kg/m^2^ 84% female	PDA—dietary and exercise software (Dietmate Pro) PDA + feedback (FB)—(PDA + FB; Dietmate Pro and customised feedback programme) Control—paper record ((PR); self-monitoring dietary intake)	Significant reductions in energy, % calories from total fat, saturated fat, and weight loss (*p* < 0.001) at 6 months There was no difference in adherence to self-monitoring and changes in dietary intake between the two PDA groups at 6 months (and were combined) There were no differences between in % weight loss in PR vs. PDA (*p* = 0.4)
2011 [61]	*n* = 210 aged 46.8 ± 9.0 years; BMI 34.01 kg/m^2^ 85% female	All groups received the same standard behavioural intervention: daily self-monitoring, group sessions, daily dietary goals, weekly exercise goals PDA—Dietmate Pro and Calcufit PDA + FB—same software plus daily tailored messages Control—PR and nutritional information book	Significant weight loss (*p* < 0.01), for all treatment groups at 6 months with no difference amongst groups Higher proportion (63%) achieved ≥5% weight loss in the PDA + FB group vs. PR group (46%; *p* = 0.04) and PDA group (49%; *p* = 0.09) Greater waist circumference decreases in PDA groups vs. PR (*p* = 0.02)
2012 [60]	*n* = 210 aged 46.8 years; BMI 27–43 kg/m^2^ 85% female	Data from [61] PDA—Dietmate Pro PDA + FB—same software plus custom algorithm for daily messages Control—PR and nutritional information book	The mean percent weight loss at 24 months was not different amongst groups; only the PDA + FB demonstrated significant weight loss (*p* = 0.02) There was no difference among the three groups in % weight change over time (*p* = 0.33) Adherence predicted weight loss at 6, 12, and 18 months
2013 [58]	*n* = 210 aged 46.8 years; BMI 27–43 kg/m^2^ 85% female	Data from [61] PDA—Dietmate Pro PDA + FB—same software plus daily feedback messages based upon the participant-recorded behaviours Control—PR and nutritional information book	Daily feedback resulted in significantly greater weight loss vs. no feedback (*p* < 0.05) Mean adherence to self-monitoring was lower for those who did not receive daily feedback than for those who did (PR and PDA 64 ± 31% vs. PDA + FB 78 ± 27%; *p* < 0.001)
2012 [59]	*n* = 210 aged 46.8 ± 9.02; BMI 34.01 ± 4.49 kg/m^2^ 85% female	Data from [61] PDA—Dietmate Pro PDA + FB—same software plus tailored daily feedback messages Control—PR and nutritional information book	Using a PDA (combined groups) had a direct effect (*p* = 0.027) on weight loss at 12 months for self-monitoring diet (*p* = 0.014) and PA (*p* = 0.014) vs. PR PDA + FB only had a significant indirect effect on weight through self-monitoring adherence to diet (*p* = 0.004) and PA (*p* = 0.002) vs. no feedback (combined groups)

**Table 3:** Study characteristics for the use of a web-based programme.

Year/Study	Participants	Digital Health Solution	Outcomes
2019 [63]	*n* = 25 Intervention aged 41.2 ± 13.9 years and BMI 34.7 ± 4.5 kg/m^2^; Control aged 52.4 ± 23.9 years and BMI 34.4 ± 4.43 kg/m^2^ 52% female	All participants received an accelerometer, blood pressure monitor, body composition scales, and were instructed by a medical doctor to follow a caloric deficit Video conference group—online curriculum for weight loss and management (one video per week, with individualised feedback) Control—scales, watch, blood pressure monitor, and provided with caloric and PA guidelines, but no weekly health coaching sessions	Intervention showed significant weight loss and % body weight loss vs. control (*p* < 0.05) Clinically significant weight loss (≥ 5%) was achieved in 69.2% of the intervention vs. 8% of the control
2020 [64]	*n* = 25 41.5 ± 13.6 years; BMI 34.6 ± 4.33 kg/m^2^ 52% female	Data from [63] Intervention—medically monitored weight management programme (weekly video-based health coaching) Control—self-guided	Rate of weight loss per week in the intervention was significantly greater vs. self-guided (*p* < 0.05) Video-based participants had 100% adherence to weekly sessions and also greater adherence to devices (*p* < 0.05)
2017 [65]	*n* = 133 aged 47 ± 11 years; BMI 35 ± 6 kg/m^2^ 100% female	5 groups: Curves Complete (CC; fitness and weight management plan, four supervised 30-min training sessions/week for 12 weeks) WW Points Plus (WW; weekly meetings at a local facility, and being able to ask questions regarding diet and exercise for feedback. Exercise was encouraged, but not required) Jenny Craig (JC; online diet-programme, received meals every 2 weeks which they supplemented with fresh fruit/vegetables and dairy, 10–15 min weekly phone sessions for dietary questions, exercise recommendations, goal setting, coping, etc. with additional online support features. The exercise was encouraged, but not required) Nutrisystem (NS; received meals every 4 weeks, focusses on the glycaemic index. Optional consultant calls whenever needed for dietary/exercise assistance. Additional online resources were available, for tracing and personnel for advice. Exercise was encouraged, but not required) Control—waiting list, instructed not to change diet or engage in PA; randomised into one of the four diet groups once completed initial stage	Significant weight loss and reduction in energy intake were found for all groups except the control. No other between-group differences existed for weight loss. Significant reduction in fat mass for CC −2.00 kg; WW −0.79 kg; JC −1.82 kg; NS −1.58 kg beginning at 4 weeks, but not for control −0.05 kg
2020 [66]	*n* = 77 Intervention aged 70.7 ± 0.25 years and BMI 29.7 ± 3.1 kg/m^2^; Control aged 71.3 ± 0.24 and BMI 28.7 ± 3.5 kg/m^2^ 50% female	Web-based exercise (WE)—10 weeks web-based weekly progressive interval training programme Control—Supervised exercise (SE); same programme in groups of 8–10 participants under fitness instructor supervision with the same volume increments. Wait-list control with a 10-week washout before WE intervention	WE had no significant effect on visceral adipose tissue (*p* = 0.5), although the SE programme did (*p* < 0.001), with no between-group differences (*p* = 0.11) Both groups significantly decreased fat mass, with a significant difference between groups (*p* = 0.042)
2020 [67]	*n* = 1298 aged 33.6; BMI 29.89 kg/m^2^ 77% female	Platform only—24-week behaviour change programme delivered using the POEmaS platform with personalised computer-delivered feedback Platform + coaching—same programme, plus 12 weeks of personalised feedback delivered online by a dietitian Control—Waiting list (non-personalised dietary and PA recommendations via e-booklet/videos)	Self-reported weight change at 12 weeks—platform only −1.14 kg, platform + coaching –1.36 kg (*p* < 0.01) vs. control −0.56 kg; no difference between intervention groups Weight change at 24 weeks—platform + coaching vs. control (*p* < 0.001) ≥5% weight loss occurred more frequently in the platform only (19.8%) and platform + coaching (15.7%) vs. waiting-list group (13%) (*p* = 0.001)
2012 [68]	*n* = 309 participants aged 42.0 ± 10.2 years; BMI 32.3 ± 4 kg/m^2^ 58% female	Basic—web-based weight-loss programme including individualised daily calorie targets for weight loss, goal settings, diaries, menu plans, tips, forums, weekly PA plans, forums, etc Enhanced—same programme with additional features, personalised e-feedback, and goals with behaviour change strategies and reminder calls Control—waiting list, asked to not engage in any other programmes, or attempt to lose weight during the intervention phase	Both intervention groups reduced their BMI compared to the control and lost significant weight (basic −2.1 kg, enhanced −3.0 kg, control 0.4 kg; *p* < 0.001). No differences were observed between basic vs. enhanced groups.
2020 [69]	*n* = 35 aged 61.54 ± 8.83 years; BMI 36.73 ± 6.84 kg/m^2^ 100% female	All participants were breast cancer survivors Intervention—SparkPeople weight loss programme (self-monitoring diet and PA, with Fitbit, weekly reminders, education, recipes/meal plans, social support; adherence monitoring at 6-months) Control—active waitlist control, the tracker only, SparkPeople at 6 months	Significant weight change at 6 months for intervention (−1.71 kg; *p* = 0.006) and greater weight loss in the control group (−2.54 kg; *p* = 0.002). No significant difference was observed between groups (*p* = 0.461) Weight loss was maintained at 12 months 33.3% of the intervention group lost ≥3% of baseline weight vs. 23.5% in the control group Number of days of food logged/week was associated with decreased waist circumference at 6 months (*p* = 0.030) and 12 months (*p* = 0.038)
2019 [70]	*n* = 76 aged 18–50 years; BMI 30–45 kg/m^2^ 66% female	Healthy Weight Programme ((HWP) 12-week programme; 10 × 1-h nutrition coaching sessions (mix of one-to-one and group classes) and 20 supervised exercise sessions with additional access to pool/gym/classes, both with 2 × progress evaluations) NHS intervention—12-week self-managed online resource, with online tools + apps and access to pool/gym. Control—gym only (no guidance or formal intervention)	Body mass, BMI, and waist circumference were significantly reduced in all groups (*p* < 0.001), with greater reductions in HWP and NHS intervention vs. gym only (*p* < 0.05)
2011 [71]	*n* = 772 Intervention aged 46.5 ± 13.5 years and BMI 31.5 ± 2.6 kg/m^2^; Control aged 48.2 ± 12.2 years and BMI 31.3 ± 2.6 kg/m^2^ 87% female	Intervention—WW, (access to weekly community-based meetings for 12 months. Monitored food intake, activity, weight change, weekly weigh-in, group discussion, behavioural counselling, motivation, forums, recipe bank) Control—standard care (national treatment guidelines), weight loss advice from a primary care professional	Both groups lost weight, but at 12 months, weight loss in the WW group was twice as much as in the control group Mean weight change at 12 months for the WW group was −5.06 ± 0.31 kg vs. −2.25 ± 0.21 kg in the control group, adjusted difference −2.77 kg (*p* < 0.0001), with the last assessment carried forward
2019 [72]	*n* = 45 median aged 61.0 years;median BMI 30.2 kg/m^2^ 100% female	WW Plus—12 × vouchers for community meetings and 16 weeks of access to digital content/tools, plus five group breast cancer-tailored dietitian-led group support sessions) WW referral—12 × vouchers for community meetings and 16 weeks of access to digital content/tools Control—No intervention, but were given a WW referral after pack at 3 months to use whenever they wished	Significant weight change for WW (−6.03 kg) and WW Plus (−3.67 kg) at 3 months (*p* < 0.001), but the control group did not change Change in weight was significant for control at 12 months (−4.22 kg) and WW (−5.11 kg; *p* < 0.05), but not long for WW Plus (−1.22 kg; *p* = 0.436) 64% WW, 56% control and 40% WW Plus lost ≥5% baseline weight by 12 months
2020 [73]	*n* = 42 aged 33.5 ± 4.0 years; BMI 32.4 ± 4.3 kg/m^2^ 100% female	Intervention—Body Balance Beyond (BBB); self-directed, designed to promote modest weight loss. Trial in women with previous gestational diabetes mellitus (last 24 months) High Personalisation Group—BBB and six individual telehealth coaching sessions via video call (20–30 min with a dietitian, weeks 2, 5, and 9) or exercise physiologist (weeks 3, 6, and 10) over the first 3 months. Text message support provided over the next 3 months. Low Personalisation Group—access to BBB Control—waiting list, no changes to diet/exercise during the intervention phase, and no attempt to lose weight.	For weight, a trend favouring the intervention groups was observed at 3 months and 6 months, although the differences among all three groups were not significant (*p* = 0.29) Sixteen women (53% of completers: high personalisation *n* = 8, low personalisation *n* = 3 and waitlist control *n* = 5) lost weight at 6 months 17% completers (*n* = 5) lost ≥5% baseline weight
2017 [74]	*n* = 271 WW online aged 55.1 ± 11.5 years and BMI 34.3 ± 3.6 kg/m^2^; WWO + ActiveLink aged 54.9 ± 11.9 years and BMI 33.8 ± 4.1 kg/m^2^; Control aged 54.9 ± 11.3 years and BMI 33.5 ± 3.3 kg/m^2^ 78% female	WW online (WWO)—12 months of online access with daily food intake and PA tracking, weekly body weight tracking via an app. PointsPlus dietary plan and tracking system WWO plus ActiveLink (WWO plus)—all resources in WWO, plus ActiveLink PA tracking device with PA goals and encouraging messages Control—online newsletters (general healthy eating and PA information) delivered weekly for 3 months, biweekly for 3 months and then monthly for 6 months	Weight loss at 3 months for WWO was −2.7 kg vs. control −1.3 kg (*p* = 0.01); neither differed from WWO plus (−2.0 kg (*p* > 0.0.5) No significant differences were observed between groups for weight loss or total dietary intake (kcal/day) at 12 months (*p* > 0.52) 24.5% WWO achieved ≥5% weight loss vs. control (9.4%) at 3 months (*p* = 0.01); neither differed from WWO plus (17.6%; *p* = 0.13–0.28) There were no significant differences among the three groups for change in daily MVPA minutes per day at 3 or 12 months (Ps > 0.17)
2020 [75]	*n* = 146 aged 58.3 ± 10.3 years; BMI 33.1 ± 4.9 kg/m^2^ 78% female	WW online programme—access to online weight management programme, personalised points system to track dietary intake for an energy deficit and increase quality with some basic diet and PA education WW + Experience Success (WW + ES)—same programme and four web-based VR sessions for training in behavioural weight-loss skills (related to the home environment, workplace, PA, and social situations at weeks 2, 4, 6, and 8)	Both groups achieved significant weight loss at 3 months (WW 2.7 ± 1.1 kg vs. WW + ES 4.2 ± 1.1 kg, both *p* < 0.001) with no difference between groups (*p* = 0.086) Greater weight loss in WW + ES at 6 months (*p* = 0.042) There was no between-group difference in the proportion of participants achieving a weight loss of ≥5% at 3 and 6 months (Ps > 0.210)
2014 [76]	*n* = 235 Intervention aged 64.7 ± 3.0 years and BMI 28.9 ± 4.7 kg/m^2^; Control aged 64.9 ± 2.8 years and BMI 29.1 ± 4.7 kg/m^2^ 41% female	Data from [77] Philips DirectLife—12-week PA programme in increasing activity with personalised goals. Accelerometer-based PA monitor, personal website, e-coach for advice for daily PA Control—waiting list, no information regarding daily PA	Intervention was significant vs. control for weight loss (*p* = 0.05), but not for BMI (*p* = 0.07) Of the 114/119 participants who completed the intervention, 50 participants (42%) successfully reached their personalised PA target (“successful” participants) Successful intervention participants lost more body weight (−2.74 ± 0.40 kg) compared to the entire intervention group (1.49 ± 0.26 kg). BMI −0.91 ± 0.13 kg/m^2^ vs. −0.29 ± 0.07 kg/m^2^ and MVPA 18.8 ± 3.9 min/day vs. −0.15 ± 1.5 min/day in the successful vs. control groups, respectively (both *p* < 0.01)
2013 [77]	*n* = 235 Intervention aged 64.7 ± 3.0 years and BMI 28.9 ± 4.7 kg/m^2^; Control aged 64.9 ± 2.8 years and BMI 29.1 ± 4.7 kg/m^2^ 41% female	Philips DirectLife—same as [76]. 12-week PA programme with monitoring/feedback, digital coaching with targets for daily activity Control—3-month waiting list, no daily activity targets	Weight decreased significantly more in the intervention group compared to controls (−1.5 kg vs. −0.8 kg, respectively, *p* = 0.046), as did waist circumference (−2.3 cm vs. −1.3 cm respectively, *p* = 0.036) and fat mass (−0.6% vs. 0.07%, respectively, *p* = 0.025). BMI did not (−0.50 ± 0.09 kg/m^2^ vs. −0.29 ± 0.07 kg/m^2^, respectively, *p* = 0.068) Daily PA increased at 13 weeks in the intervention group by 46 ± 7% (*p* < 0.001) vs. control 12 ± 3% (*p* < 0.001) by the ankle accelerometer and by 11 ± 3% (*p* < 0.001) vs. control 5 ± 2% (*p* = 0.027) by the wrist accelerometer

**Table 4:** Study characteristics for the use of tailored text/phone calls.

Year/Study	Participants	Digital Health Solution	Outcomes
2016 [78]	*n* = 228 Intervention aged 55.5 ± 12.3 years and BMI 29.3 ± 5.8 kg/m^2^; Control aged 51.2 ± 11.9 years and BMI 29.6 ± 6.3 kg/m^2^ 67% female	Intervention—Get Healthy, Stay Healthy (GHSH; via individually tailored text messages, with data collected during two telephone calls with goal setting and targets consistent with national guidelines) Control—brief written feedback of results following each assessment, no other contact	Significant intervention effects on weight loss at 6-months (*p* = 0.003), moderate PA sessions/week (*p* = 0.008), and accelerometer-assessed MVPA (*p* = 0.007) No difference in waist circumference, dietary outcomes, and other PA outcomes between groups
2015 [79]	*n* = 196 males Intervention aged 41.02 ± 6.82 years and BMI 28.0 ± 2.15 kg/m^2^; Control aged 41.55 ± 6.98 years and BMI 27.6 ± 2.5 kg/m^2^ 0% female	Intervention—6-month programme including tailored text message reminders every other day, plus four offline education sessions and brief counselling with monthly weight checks by nurses for weight control Control—four offline education sessions and brief counselling with monthly weight checks by nurses about weight control	Both groups significantly reduced their body weight compared with baseline (1.71 kg intervention vs. 1.56 kg control). At 1 month, weight loss was significant between groups (*p* = 0.01) but at 6 months, weight loss between groups was not significant (*p* = 0.78) There was no significant difference between groups for % body fat (*p* = 0.60) and PA min/wk (*p* = 0.14) at 6 months
2021 [80]	*n* = 459 aged 23.3 ± 4.4 years; BMI 31.2 ± 4.4 kg/m^2^ 79% female	Targeted—Facebook content and generic daily text messages to reinforce self-monitoring and provide tips. The intervention was weight loss focussed including content adapted from the Diabetes Prevention Programme with calorie, weight loss, and PA goals Tailored—Facebook content and 6 tailored text messages/wk—specific prompts for self-monitoring weight, PA, with additional personal and generic messages for feedback, tips and reminders. Intervention was weight loss focussed including content adapted from the Diabetes Prevention Programme with calorie, weight loss, and PA goals Control—Facebook delivery component. Wellness content related to healthy body weight (e.g., sleep, stress, body image). The content was educational rather than focussing on specific behaviour change	No overall effect of the treatment group on 6, 12, and 18-month weight loss Subset engagement analysis: engagement in ≥66% of the personalised intervention (Tailored) lost more weight vs. the control group at 6 months (*p* = 0.004), with the trend continuing at 12 months (*p* = 0.05), but disappearing by 18 months Participants in the lowest BMI category (25–27.5 kg/m^2^) in the Tailored group lost 2.27 kg more than the control (*p* = 0.006) and those in the Targeted group lost 1.72 kg more than the control (*p* = 0.02) after adjusting for covariates at 6 months
2014 [81]	*n* = 185 aged 35.4 ± 5.5 years; BMI 30.2 ± 2.5 kg/m^2^ 100% female	Shape programme (12 months)—behaviour change goals to promote weight loss, self-monitoring via weekly interactive voice response (IVR) calls, tailored skills training materials, monthly dietitian calls, 12-month YMCA membership Control—usual care (routine standard of care from providers)	IVR completion rate at 12 months was 71.6 ± 28.1% (weekly range from 52–96%) and 52% had an IVR completion rate of ≥80% with two-thirds completing at least 60% of IVR calls At 12 months, IVR call completion was significantly correlated with weight loss (*p* = 0.04) and those with ≥80% IVR completion rate had greater weight loss vs. those who had <80% IVR completion rate (*p* = 0.01), with similar outcomes for BMI (mean difference −0.94 kg m^2^; *p* = 0.009) (−0.70 ± 0.25 kg/m^2^ ≥80% IVR completion rate vs. 0.25 ± 0.25 kg/m^2^ <80% IVR completion rate)
2015 [82]	*n* = 67 aged 48.2 ± 11.7 years; BMI 31.0 ± 3.7 kg/m^2^ 91% female	Intervention—self-monitoring with Fitbit One tracker + SMS-based PA prompts Control—self-monitoring with Fitbit One only	Significant between-group differences in PA change from baseline to week 1 for steps (*p* = 0.01), fairly/very active minutes (*p* < 0.01), and total active minutes (*p* = 0.02), but these changes in PA were the short-term and not maintained through weeks 2–6 Significant within-group increase of +4.3 ±2.0 min/week of MVPA from baseline to 6 weeks follow up in the control group (*p* = 0.04), but no group differences across PA levels
2018 [83]	*n* = 191 aged 49 ± 10.5 years; BMI 36.7 ± 4.3 kg/m^2^ 92% female	Participants had lost at least 5 kg during the first 4–6 months in the WW programme and then recruited to one of three arms for 6 months, followed by passive monitoring for months 7–12; Direct—WW + direct monetary incentive + daily self-weighing and text messaging feedback Lottery—WW + additional lottery-based monetary incentive + daily self-weighing and text messaging feedback Control—daily self-weighing and text messaging feedback	Weight loss pre-trial before randomisation was 11.4 ± 4.7 kg Maintenance of weight loss occurred across all arms (direct −2.8 ± 5.8 kg, lottery −3.0 ± 5.8 kg, control −1.4 ± 5.8 kg), significant in the two intervention arms (*p* < 0.001) but not control. There was no significant difference between arms for weight loss at 12 months (*p >* 0.1) and changes in self-reported PA and eating behaviours did not differ across arms Participants who maintained their weight loss (defined as gaining ≤1.36 kg) at 6 months—lottery 79%, direct 76%, control 67% (*p* > 0.1); 12 months *p* > 0.1

## Data Availability

No new data were created or analysed in this study. Data sharing is not applicable to this article.

## Abbreviations

Body Mass Index (BMI); Physical Activity (PA); National Health Service (NHS); Cell Phone Intervention For You (CITY); Myfitnesspal (MFP); Weight Watchers (WW); Personal Digital Assistant (PDA); Intensive Counselling (IC); Weight Management Programme (WMP); Beyond The Scale (BTS); Mobile Phone-Based Diabetes Prevention Programme (mDPP); Be Positive Be Health (BPBH); Videoconferencing (VC); In-Person (IP); Primary Care Physician (PCP); Total Body Weight (TBW); Mobile Health And Diabetes (Mdiab); Weight-Loss Maintainers (WLM); Moderate To Vigorous Physical Activity (MVPA); Personal Digital Assistant (PDA); Tailored Feedback (TF); Feedback (FB); Paper Record (PR); Curves Complete (CC); Jenny Craig (JC); Nutrisystem (NS); Web-Based Exercise (WE); Supervised Exercise (SE); Healthy Weight Programme (HWP); Body Balance Beyond (BBB); WW Online (WWO); WWO Plus Activelink (WWO Plus); WW + Experience Success (WW + ES); Visceral Adipose Tissue (VAT); Get Healthy; Stay Healthy (GHSH); Interactive Voice Response (IVR).
